# Neutron reflectometry under high shear in narrow gap for tribology study

**DOI:** 10.1038/s41598-023-45161-9

**Published:** 2023-10-25

**Authors:** Naoki Yamashita, Tomoko Hirayama, Masahiro Hino, Norifumi L. Yamada

**Affiliations:** 1 Department of Mechanical Engineering and Science, Graduate School of Engineering, Kyoto University, Katsura, Nishikyo-ku, Kyoto, 615-8540 Japan; 2 Institute for Integrated Radiation and Nuclear Science, Kyoto University, Kumatori, Osaka 590-0494 Japan; 3 Institute of Materials Structure Science, High Energy Accelerator Research Organization, 1-1 Oho, Tsukuba, Ibaraki 305-0801 Japan

**Keywords:** Mechanical engineering, Applied physics

## Abstract

An *operando* analysis method has been established for evaluating the interfacial structure of an adsorbed layer formed by an additive on a metal surface under fluid lubricated conditions. A parallel-face narrow gap viscometer installed in an energy-resolved neutron reflectometer is used to evaluate the change in the interfacial structure under high shear. The viscometer was designed to operate at a high shear rate while maintaining a µm-order constant gap between two parallel surfaces. When an additive-free base oil was sandwiched in the gap, the neutron reflectivity profiles without and with upper surface rotation were the same. This demonstrates that the reflectivity profiles can be accurately measured regardless of whether the upper surface is rotated. When a base oil containing a polymethacrylate-based additive was sandwiched in the gap, both the thickness and density of the adsorbed additive layer in the rotation (shear field) condition were lower than those in the non-rotation (static) condition. This demonstrates that the proposed method can be used to analyse the structural changes in the adsorbed layer formed by an oil additive on a surface. This combination of a neutron reflectometer and narrow gap viscometer is a promising approach to near-future tribological studies.

## Introduction

Machine components are normally operated under oil lubricated conditions to prevent damage due to direct contact between opposing sliding surfaces. Many studies of mechanical sliding surfaces under lubricated conditions have been conducted in the field of tribology. The friction behaviours of sliding surfaces under lubricated condition can be divided into three states: boundary lubrication, mixed lubrication, and fluid lubrication. Under fluid lubricated conditions, the shear resistance of the oil in the gap between the surfaces is the dominant factor in friction force because the two surfaces are completely separated by the oil film. Friction loss can be suppressed by using a low-viscosity oil to reduce fluid resistance. However, since low-viscosity oils make the oil film thinner and reduce the gap between the surfaces, the risk of seizure is increased as is the friction on and wear of the surfaces. The trend toward using low-viscosity oil to reduce friction loss has made lubricant additives in protecting surfaces particularly important. It is thus essential to evaluate the characteristics of the adsorbed layer formed in a narrow gap by additives under lubricated conditions.

There are several papers on how the presence of additive adsorption layers exhibit friction behaviour under elastohydrodynamic (EHL) lubrication. For examples, the entrainment speed dependence of the friction coefficient and the oil film thickness of lubricants containing various additives such as fatty acids and polymer additives have been investigated^1–5^. In contrast, few studies have investigated the effect of the adsorbed layer formed on metal surfaces by lubricant additives under fluid lubricated conditions^6,7^, and the effect of shear on the adsorbed additive layer structure remains unclear.

In this study, neutron reflectometry was used to investigate the structure of adsorbed additive layers under high shear rate simulating fluid lubricated conditions. Neutron reflectometry is becoming widely used in the field of tribology, but it is mainly used to evaluate the thickness and density of adsorbed additive layers in a static state^8–10^. For example, fatty acids such as palmitic and oleic acids were found to form a nanoscale adsorbed layer on Fe, Cu, and diamond-like carbon, with thicknesses roughly equivalent to their molecular chain lengths^11–14^. Evaluation of oil-soluble polymeric friction modifiers clarified the temperature dependence of the thickness and density of the adsorbed layer formed under static conditions^1^. In contrast, few *operando* analyses in a shear field have been conducted^15^, and the change in the structure of the adsorbed layer formed by an additive at the sliding surfaces has not been clarified.

To obtain a clear neutron reflectivity profile, it is essential to use a substrate coated with a smooth thin metal film. It is thus common to deposit a metal film with a thickness of 30–50 nm on a mirror-polished silicon block using a metal deposition system^16,17^. However, damage to the metal film due to friction during neutron reflectometry causes *operando* analysis to fail. Therefore, when Armstrong et al. analysed the structure of an adsorbed layer of glycerol monooleate (GMO) under shear conditions, they set the gap between the roller and flat disk to as much as 200 µm^15^. Reflectometry experiments have also been performed with a commercially available rheometer installed in a reflectometer, but the minimum gap between the cone and plate is typically on the order of 100 µm, which does not correspond to the typical oil film thickness under tribological conditions, and the shear rate was typically only around 10^3^ s^−1^^15,18–21^.

The objective of this study was to establish an *operando* analysis method for evaluating the interfacial structure of an adsorbed layer formed by an additive on a metal surface under fluid lubrication conditions in which a non-contact condition was maintained with a few micrometres gap. To achieve this, we developed an analytical method that combines the use of a neutron reflectometer with that of a previously developed parallel-disk viscometer with a narrow gap that can measure the shear force of oil film between two parallel surfaces. Experiments using the narrow gap viscometer in the past demonstrated that interfacial slip occurs on the adsorbed layer formed by a fatty acid. There was no direct contact between the two sliding surfaces, and the adsorbed layer reduced shear force under fluid lubrication conditions even when the gap was less than several µm^6^.

The feasibility of interfacial analysis by installing the narrow gap viscometer in a neutron reflectometer was tested in this study. A high shear force was applied while maintaining a narrow gap between two parallel surfaces sandwiching lubricant and entering a neutron beam onto the interface. First, an experiment using an additive-free base oil was conducted to check for any change in the reflectivity profile under static and shear conditions. Next, the structure of the adsorbed layer of a polymer additive formed on a Cu layer under high shear in a narrow gap was evaluated.

## Parallel-face narrow gap viscometer

### Apparatus configuration

**Figure 1:**
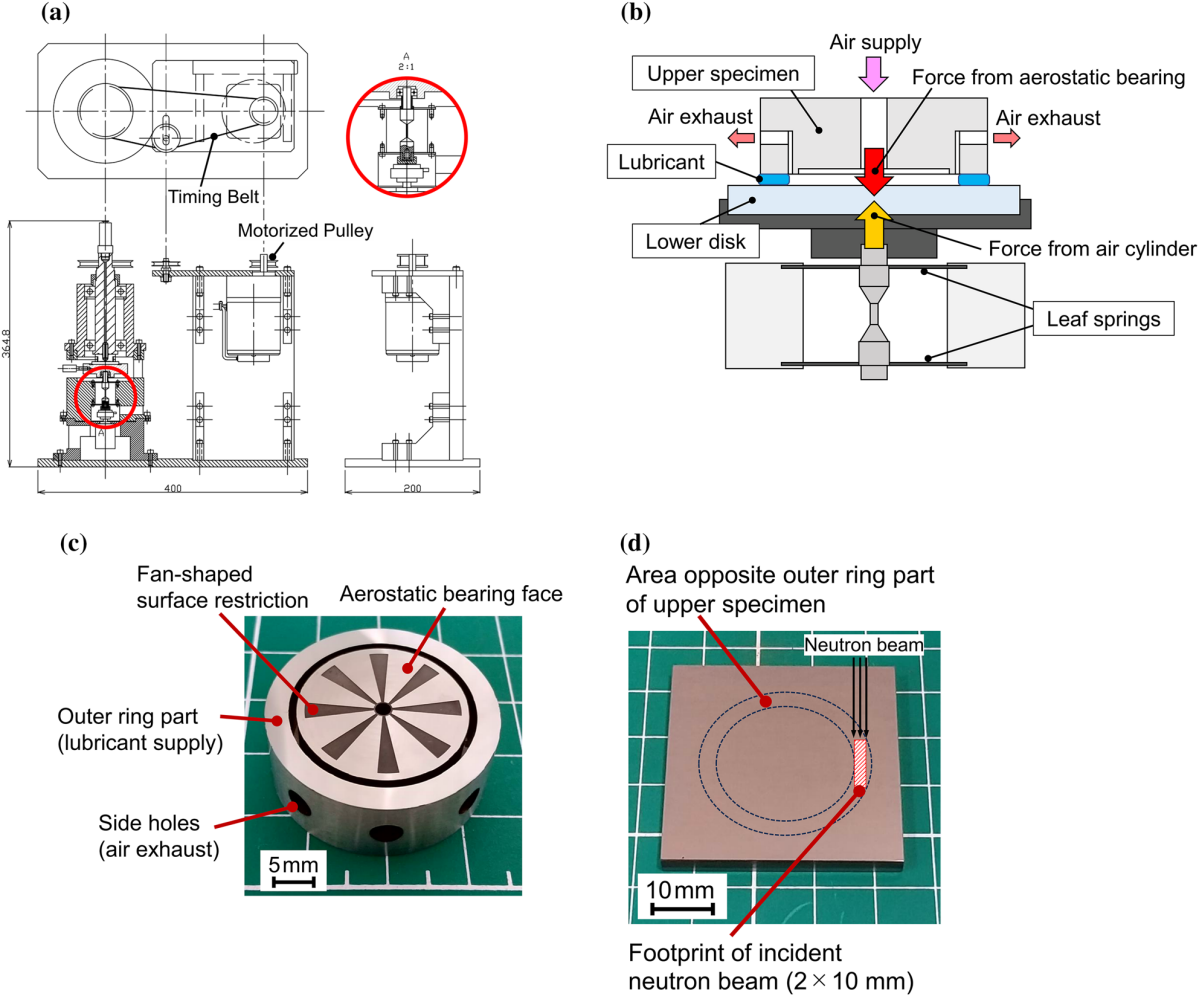
(**a**) Schematic diagram of parallel-face viscometer with narrow gap; (**b**) schematic illustration of viscometer components, (**c**) picture of upper specimen, (**d**) picture of lower disk.

A schematic illustration of the previously designed narrow gap viscometer and its components are shown in Fig. 1a–d^6^. The parallel-face configuration is marked by the solid red circle in Fig. 1a and its details is shown in Fig. 1b. A narrow gap is configured between the upper specimen (Fig. 1c) and the lower disk (Fig. 1d). The upper specimen is made of stainless steel (inner diameter: 24 mm; outer diameter: 30 mm) and contains an aerostatic bearing pad in the centre. Sample lubricant was supplied only between the flat outer ring part of the upper specimen and the lower disk, as shown in Fig. 1b. The upper specimen was rotated using a motorized pulley and timing belt arrangement. The aerostatic bearing enables the upper specimen to rotate while a virtually constant gap (~ several µm) is maintained between the upper specimen and the lower disk. The gap remains stable at a constant value, which depends on the supplied air pressures, regardless of the rotational speed of the upper specimen.

The lower disk is a mirror-polished silicon block (40 × 40 × t4 mm) mounted on a holder supported by two specially shaped leaf springs that are rigid in the axial direction while flexible in the inclined direction. The two surfaces can thus remain parallel without any contact owing to the tilting stiffness of the aerostatic bearing. In the previous study^6^, a load cell was used to measure the rotational torque generated by the shear force, but it was not used in this study due to the space limitation imposed by the installation of the neutron reflectometer.

### Gap control

Gap control is explained in detail in our previous report^6^, so only a brief description is given here. As shown in Fig. 1c, the central part of the upper specimen has the aerostatic bearing (outer diameter: 22 mm), with its surface recessed approximately 5 µm. The bearing surface has fan-shaped surface restrictions and is supplied with air through a central hole (diameter: 2 mm). The supplied air is prevented from leaking through the gap by the lubricant sandwiched between the flat outer ring part of the upper specimen and the lower disk; it is eventually exhausted through holes on the side wall of the upper specimen. A downward force is generated by adjusting the pressure of the air supplied to the aerostatic bearing, and the gap between the two parallel surfaces is determined by the equilibrium achieved by applying upward force generated by an air cylinder under the lower disk.

The load capacity of the aerostatic bearing is calculated by using the compressible Reynolds equation to theoretically predict the gap between the aerostatic bearing and the lower disk surface. The force generated by the pressure of the supplied air was measured beforehand by setting a load cell between the lower disk and the air cylinder to calibrate the relationship between the supplied air pressure and the gap. The gap varies only with the air pressure supplied to the aerostatic bearing; it does not depend on the viscosity of the sample lubricant or the rotational speed. That is, the gap remains constant even during rotation.

## Feasibility evaluation

### Overview of neutron reflectometry

In neutron reflectometry, the thickness and density of an adsorbed layer formed by an additive on metal film can be evaluated since the layer structure at the solid–liquid interface is clearly distinguished by replacing the hydrogen atoms of either the base oil or the additive with deuterium atoms. In our experiment, the target surface was the top of the lower disk. A Cu layer with a thickness of 30 nm was previously formed on the lower disk surface by ion beam sputtering (KUR-IBS system^22^). Copper is a common industrial material used in mechanical elements such as sliding bearings. Unlike Fe, Cu is nonmagnetic, which enables simple and accurate analysis. The surface roughness of the Cu layer measured by atomic force microscopy was Ra ~ 0.5 nm.

A time-of-flight energy-resolved neutron reflectometer SOFIA^23,24^ installed at BL16 of the Materials and Life Science Experimental Facility in the Japan Proton Accelerator Research Complex (J-PARC MLF) was used for the feasibility evaluation and main experiments. The narrow gap viscometer was set on the reflectometer, and a neutron beam was incident from the side wall of the lower Si disk to obtain the reflectivity profile at the interface between the top of the lower disk with the thin Cu layer and lubricant.

**Figure 2:**
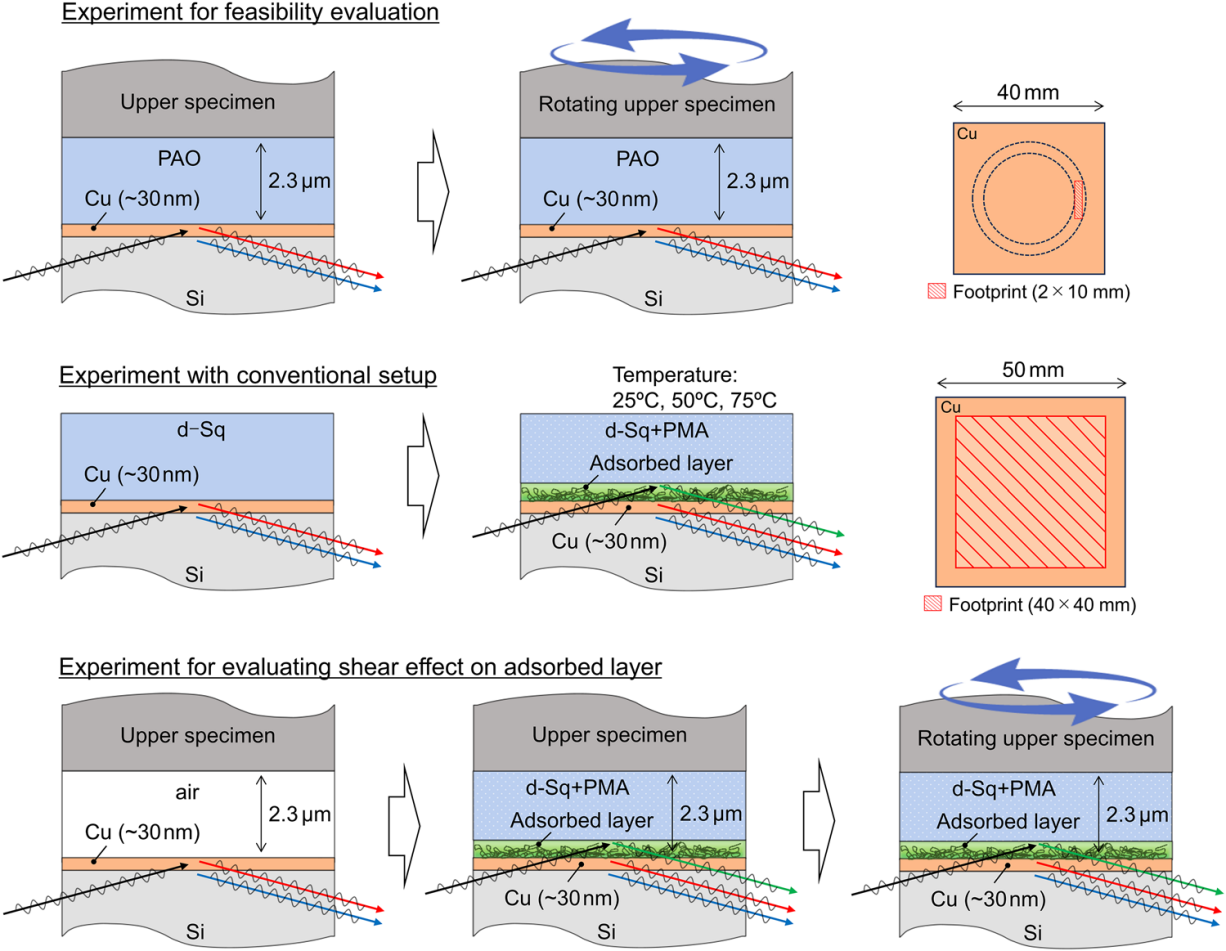
Setup and procedure for neutron reflectometry experiments.

A schematic illustration of the measurement is shown in Fig. 2. A neutron beam was incident on the sample at a small angle *θ*, and the ratio of all incident neutrons to those reflected at the interface was defined as reflectivity *R*. In the reflectivity profiles, scattering vector *Q*_z_, calculated as follows, was set in the horizontal axis.

1 $$Q_{{\text{z}}} = 4\pi {\text{sin}}\theta /\lambda ,$$

where *λ* is the wavelength of the neutron beam. The profiles depended on the layer structure of the target interface. Fitting analysis was performed using MOTOFIT analysis software^25^. Each material has a specific scattering length density (SLD), which enabled identification of the layer structure formed on top of the lower disk. The SLD values relevant to this study are listed in Table 1.

**Table 1:** Theoretical SLD values for materials relevant to this study.

Material	SLD [× 10^–6^ Å^–2^]
Si	2.1
SiO_2_	3.5
Cu	6.6
Cu_2_O	5.4
CuO	6.5
Cu(OH)_2_	2.5
PAO	–0.4
d-Sq	7.1
d-Sq + mineral oil	6.3
PMA	0.0

### Neutron reflectometry in high shear field with additive-free base oil

To determine the feasibility of the proposed analysis method, a neutron reflectometry experiment was conducted using the narrow gap viscometer. Poly-alpha-olefin (PAO), a typical base oil, was sandwiched between the flat outer ring part of the upper specimen and the lower disk. The air pressure supplied to the aerostatic bearing through the upper specimen was 0.27 MPa, and the air pressure to the air cylinder below the lower disk was 0.36 MPa, then the resulting gap was 2.3 µm. The footprint of the incident neutron beam was set to 2 × 10 mm at any incident angle, as shown in Fig. 1d, so that it did not extend beyond the lubricant sandwiched area between the flat outer ring part of the upper specimen and the lower disk. This footprint is comparatively small, about 1/100 that in reflectivity measurements under static conditions using a conventional setup, as described in “Analysis of adsorbed additive layer in static state” section. As a result, the reflected neutron count was about 150/s, which is comparatively low, so the time required for obtaining each reflectivity profile was ~ 8 h.

First, the refractivity profile was obtained under static conditions; that is, the upper specimen was not rotated. Two reflectivity profiles were obtained using neutron incident angles of 0.4° and 0.8°. They were then combined to form a single reflectivity profile. Next, the same measurements were conducted under shear conditions; that is, the upper specimen was rotated at a constant speed. The rotation speed was set to 100 rpm, so the shear rate was 6.1 × 10^4^ s^−1^.

### Results of feasibility test

**Figure 3:**
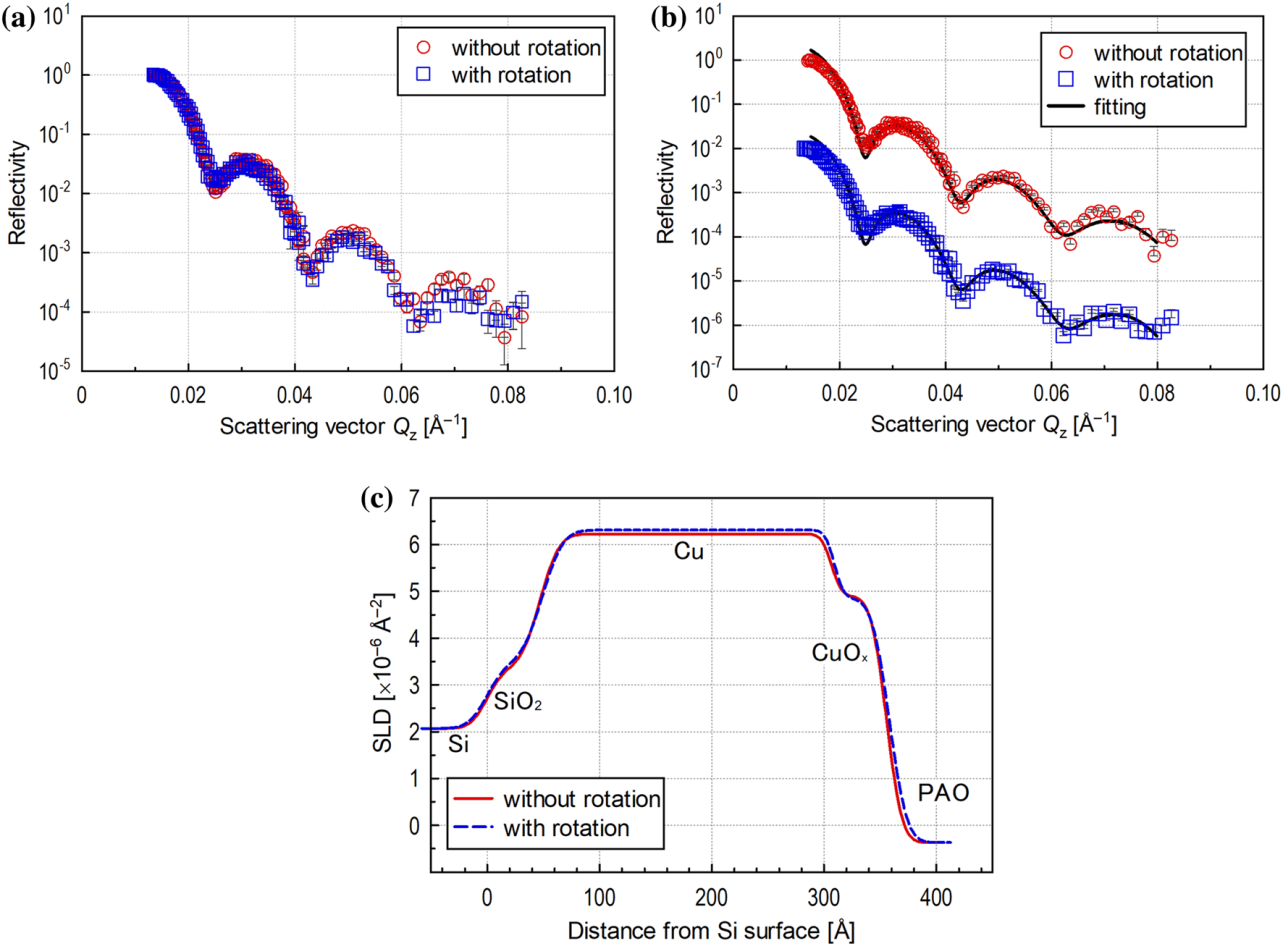
(**a**) Neutron reflectivity profiles without and with upper specimen rotation; (**b**) neutron reflectivity profiles offset for visibility with optimum fitting line; (**c**) SLD profiles obtained by optimum fitting for reflectivity profiles without and with upper specimen rotation.

Figure 3a and 3b show the reflectivity profiles obtained without and with upper specimen rotation while maintaining the 2.3 µm gap. Figure 3a shows the profiles overlaid, and Fig. 3b shows them offset in the vertical axis direction for better visibility. As shown in Fig. 3a, the profiles were almost the same. The interfacial fringes on the profiles did not change, indicating that the difference in reflectivity profiles between without and with rotation is negligible.

Figure 3c shows the SLD profiles obtained by fitting analysis for the reflectivity profiles shown in Fig. 3b. The range of scattering vector *Q*_z_ was smaller, and the errors in each measurement plot were larger than those with the conventional setup described in “Analysis of adsorbed additive layer in static state” section. Detailed structural analysis of the Cu_2_O, CuO, and Cu(OH)_2_ in the Cu thin layer on the lower Si disk that may form naturally after deposition of the Cu layer could not be performed. This is because reflectivity profiles with sufficient information could not be obtained due to insufficient measurement time resulting from the small footprint and low neutron reflection rate. Fitting analysis was thus performed using a model in which a single layer of Cu oxide (CuO_x_) was assumed to exist in a different state than the bulk Cu. The thicknesses of the SiO_2_, Cu, and CuO_x_ layers in measurements without upper specimen rotation were estimated to be 4.7 nm, 26.0 nm, and 4.9 nm, respectively, whereas those with rotation were estimated to be 4.9 nm, 26.0 nm, and 4.9 nm, respectively. The thicknesses of the Cu and CuO_x_ layers were about 31 nm, which corresponds to the target value for Cu deposition by sputtering. This indicates that the analysis was performed correctly.

These results demonstrate that rotation of the upper specimen had little effect on the reflectivity profile, which means that the interfacial structure on the lower disk can be analysed precisely. In addition, they demonstrate that the combination of the neutron reflectometer and the narrow gap viscometer, which does not damage a thin Cu layer on top of the lower disk during long-term measurement, enables the measurement of neutron reflectivity at a higher shear rate than in previous studies.

## Interfacial analysis of adsorbed polymer layer

### Sample lubricant

The use of polymethacrylate (PMA)-based additives has become widespread, especially due to the growing use of lower viscosity oils. As the sample lubricant for our study, a PMA-based additive was prepared and added to deuterated squalane (d-Sq), a non-polar solvent, at a concentration of 2%. This lubricant contains slightly less than 10% mineral oil, which is used for dilution to facilitate handling of the highly viscous PMA. The PMA has a molecular weight of ~ 430,000 g/mol and is composed of methacrylate monomers with short alkyl chains, such as methyl methacrylate, and methacrylate monomers with branched alkyl chains. The relatively high content of methacrylate with short alkyl chains makes it highly adsorptive to metal surfaces. The radius of gyration of the PMA in solvent is estimated to be around 10 nm based on its molecular weight, while it has some variation depending on the compatibility of the base oil and PMA.

### Analysis of adsorbed additive layer in static state

A precise analysis of the adsorbed layer structure formed by the PMA in the static state was carried out using a previously proven method with a liquid cell^1,3,10,13^. A mirror-polished Si block (50 × 50 × t10 mm) covered with a thin sputtered Cu layer was used. First, the neutron reflectivity from the Cu layer was obtained after setting the Si block in a sample holder and immersing it in d-Sq without PMA. After the d-Sq was replaced with the lubricant containing the PMA, the neutron reflectivity was measured again at room temperature (~ 25 °C) to evaluate the structure of the adsorbed layer formed by the PMA on the Cu layer surface. The temperature dependence of the structure of the adsorbed layer was also evaluated by additional measurements after heating at 50 °C and 75 °C for 30 min. The reflectivity profiles were then obtained at the incident angles of 0.3°, 0.6°. and 1.2° with the beam footprint of 40 × 40 mm at any incident angle, and they were then combined to form a single reflectivity profile. The time required for obtaining this single reflectivity profile was ~ 15 min.

**Figure 4:**
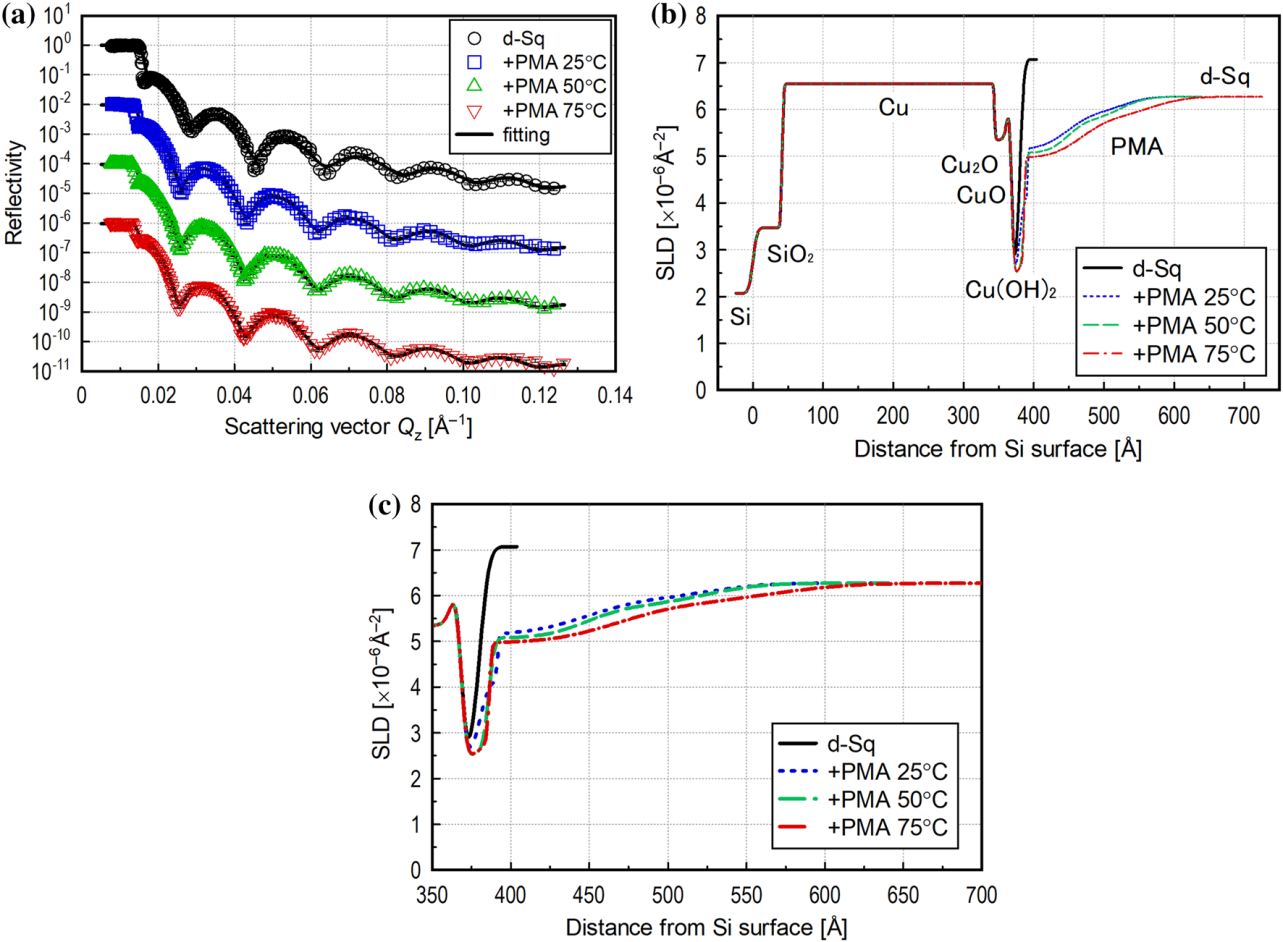
(**a**) Neutron reflectivity profile with offset for visibility with optimum fitting line in static state; (**b**) SLD profiles obtained by optimized fitting analysis in static state for evaluating structure of adsorbed layer of PMA additive; (**c**) SLD profiles zoomed in for area of adsorbed layer.

The reflectivity profiles obtained in the static state are shown in Fig. 4a. The results are shifted in the vertical axis direction for better visibility, and the optimum solutions obtained by fitting analysis are shown as solid lines. The SLD profiles obtained by fitting analysis are shown in Fig. 4b and c. The range of scattering vector *Q*_z_ was wide, and the error in each plot is small since a sufficient number of reflected neutrons was counted. Therefore, a precise analysis could be performed by modelling the formation of Cu_2_O, CuO, and Cu(OH)_2_ layers on top of the Cu layer.

The SLD of the PMA used in this research was almost 0.0 × 10^–6^ Å^–2^, whereas that of the base oil (d-Sq + mineral oil for dilution) was 6.3 × 10^–6^ Å^–2^. Therefore, an adsorbed layer with a lower SLD contains PMA with a higher density, and an adsorbed layer with a higher SLD has more d-Sq penetration. Fitting analysis showed that the adsorbed PMA layer on the Cu layer consisted of a higher density layer with a thickness of ~ 2 nm and a lower density layer with a thickness of ~ 20 nm on the higher density layer (Fig. 4c). There was a tendency for the thickness of the adsorbed layer to increase slightly with the temperature, which is consistent with the behaviour observed in a previous study^1^. However, the differences between the reflectivity profiles obtained at the three temperatures were not significant, suggesting that the PMA used was in a swollen state even at 25 °C due to its high compatibility with d-Sq.

### Analysis of adsorbed additive layer in high shear field

For neutron reflectometry measurement of the adsorbed PMA layer in a high shear field, a new lower Si disk with a surface Cu layer was prepared. The upper and lower supplied air pressures in the narrow gap viscometer were again set to 0.27 and 0.36 MPa, respectively, to create a 2.3-µm gap between the upper specimen and the lower disk. This gap was maintained during the measurement by the aerostatic bearing. Before the experiment, 40 µL of d-Sq containing PMA was supplied into the gap. First, the reflectivity profile was obtained without rotation to evaluate the structure of the polymer adsorbed layer that formed on the Cu layer surface in the static condition. Next, the upper specimen was rotated, and the reflectivity profile was again obtained. The rotation speed was 100 rpm, so the shear rate was 6.1 × 10^4^ s^−1^. The footprint was 2 × 10 mm, and the reflectivity profile was obtained with an incident angle of 0.6° only. The time required for obtaining one reflectivity profile was ~ 8 h.

**Figure 5:**
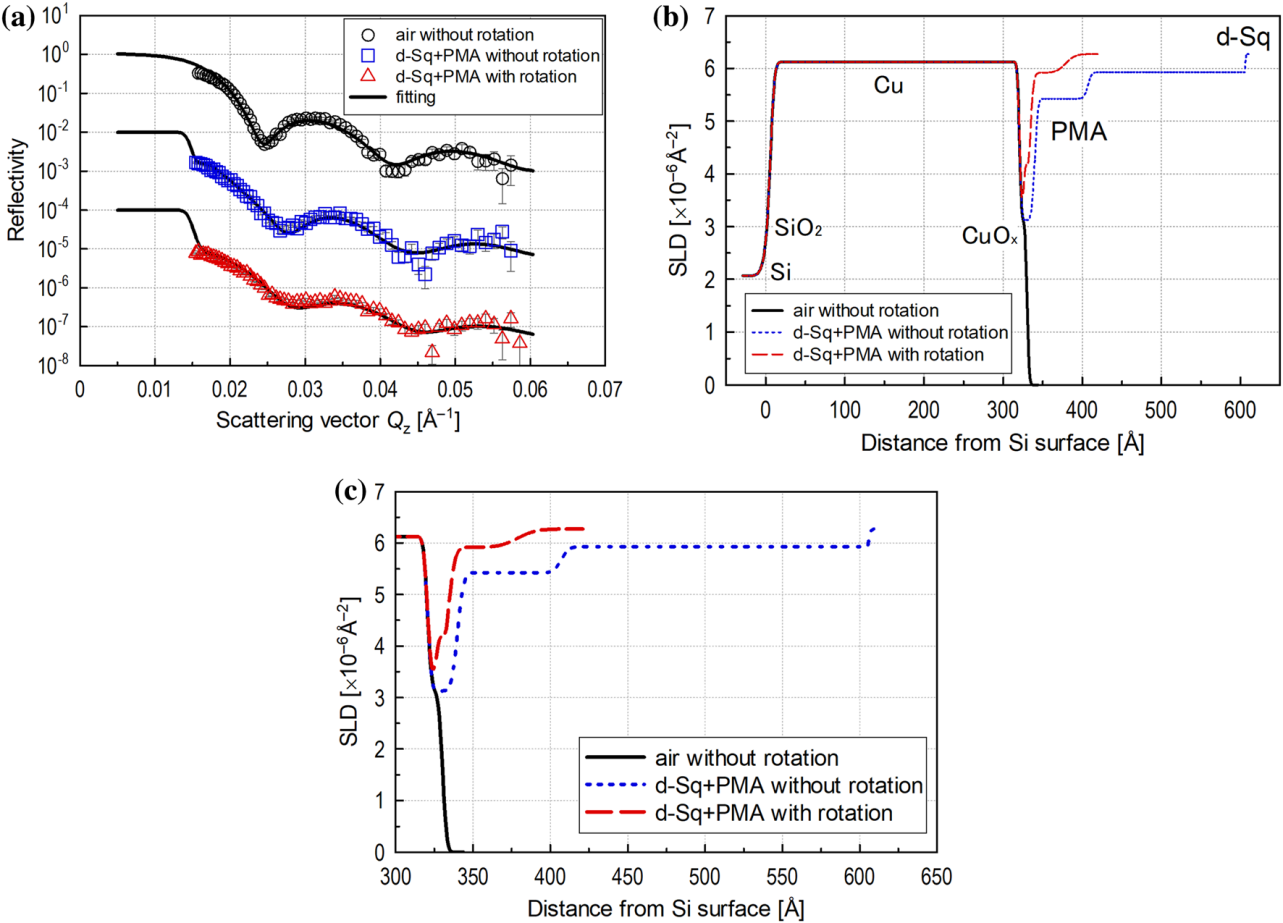
(**a**) Neutron reflectivity profile with offset for visibility with optimum fitting line for measurements with narrow gap viscometer; (**b**) SLD profiles obtained by optimum fitting for measurements with the narrow gap viscometer; (**c**) SLD profiles zoomed in for area of adsorbed PMA layer.

The obtained reflectivity profiles are shown in Fig. 5a. This graph shows the results in air (top), and with lubricant under static (middle) and shear (bottom) conditions with fitting lines and offsets in the vertical axis direction for better visibility. The appearance of the fitting line for in air in the small *Q*_z_ range (< 0.015 Å^–1^) differs from the two in lubricant because total neutron reflection does not occur in air measurements.

The SLD profiles obtained by the fitting analysis are shown in Fig. 5b and c. The surface structure of the Cu layer was first determined by fitting analysis for the reflectivity profile measured in air without lubricant. The Cu oxide layer could not be precisely analysed due to the small footprint, but the thicknesses of the Cu and CuO_x_ layers obtained by the fitting were close to the values described in “Analysis of adsorbed additive layer in static state” section, indicating that the analysis was generally appropriate.

Next, the structural changes in the adsorbed PMA layer without and with rotation of the upper specimen were evaluated using a model in which the state of the Cu layer determined by measurement in air was assumed to remain unchanged, and the adsorbed PMA layer was assumed to form on top of the Cu layer. The SLD profile obtained by fitting analysis of the reflectivity profile in the static condition shows that the adsorbed layer had a thin higher density part and a thick lower density part, similar to the findings presented in “Analysis of adsorbed additive layer in static state” section. This demonstrates that the structure of the adsorbed additive layer can be analysed well in neutron reflectometry even with the narrow gap viscometer setup.

As shown in Fig. 5a, the reflectivity profile with rotation clearly differed from that without rotation, and, as shown in Fig. 5c, the SLD of the adsorbed additive layer increased with rotation. This indicates that a large amount of d-Sq with a higher SLD penetrated the adsorbed PMA layer, meaning that the density of the adsorbed layer became lower in the high shear condition. The distance from the top surface of the Cu layer to the area where the SLD reached that of the bulk lubricant (6.3 × 10^–6^ Å^–2^) was shorter than that measured in the static condition, indicating that the thickness of the adsorbed layer also became thinner in the shear field. Although the temperature at the interface was not controlled, this difference is not temperature-dependent, as shown by the finding that the differences between the reflectivity profiles obtained at three temperatures were not significant (“Analysis of adsorbed additive layer in static state” section). In general, certain polymers are well known to align in lubricant due to the shear thinning effect. If the adsorbed PMA layer in our experiment aligned by shear, the PMA layer thickness should decrease, whereas the density of the PMA layer should increase. However, the density of the PMA layer also decreased. Therefore, it is highly likely that some of the PMA molecules that adsorbed onto the Cu surface desorbed due to the shear force. These results indicate that the adsorbed PMA layer could not maintain the fully adsorbed state in the shear condition and that the number of adsorbed PMA molecules in the shear field was less than that in the static condition. Estimating the change in the volume of adsorbed PMA molecules from the SLD profile, it was found that 74% of the PMA molecules adsorbed in the static condition were desorbed by shear.

## Summary

A parallel-face narrow gap viscometer was installed in an energy-resolved neutron reflectometer and used to investigate the adsorbed additive layer structure under high shear rate conditions. Experimental results demonstrated that sufficient reflectivity profiles can be obtained to allow analysis even with the narrow gap viscometer regardless of whether there is rotational operation. Measurements with the viscometer sandwiching lubricant containing PMA additive demonstrated that both the thickness and density of the adsorbed PMA layer under high shear became lower than those under static condition, indicating that the polymer additive desorbed from the Cu surface, likely due to the high shear force. This finding will be useful in designing lubricant additives that can function under a wide range of lubricating and high shear rate conditions. The combination of a neutron reflectometer and the narrow gap viscometer is thus a promising approach to near-future tribological studies.

## Data Availability

The datasets in this study are available from the corresponding author on reasonable request.
